# Using Microdosing to Induct Patients Into a Long-Acting Injectable Buprenorphine Depot Medication in Low Threshold Community Settings: A Case Study

**DOI:** 10.3389/fphar.2021.631784

**Published:** 2021-03-23

**Authors:** Joseph Tay Wee Teck, Alexander Baldacchino, Lauren Gibson, Con Lafferty

**Affiliations:** ^1^ School of Medicine, Population and Behavioural Science Division, University of St Andrews, St Andrews, United Kingdom; ^2^ Harm Reduction Team, NHS Lothian, Edinburgh, United Kingdom; ^3^ NHS Fife Addiction Services, Cameron Hospital, Fife, United Kingdom

**Keywords:** opioid use disorder, buprenorphine, microdosing, drug related deaths, outreach

## Abstract

Healthcare innovation has never been more important as it is now when the world is facing up to the unprecedented challenges brought by the COVID-19 pandemic. Within addictions services in Scotland, the priority has been to tackle our rising drug related death rate by maintaining and improving access to treatment while protecting frontline workers and managing operational challenges as a result of the pandemic. We present here a case study of five patients with opioid use disorder whose treatment represents a confluence of three important Medication Assisted Treatment (MAT) service innovations. The first was a low threshold drop in and outreach MAT service to rapidly and safely initiate opiate replacement therapy (ORT). The second was the provision of a microdosing regimen to enable same day induction to oral buprenorphine while minimizing the risk of precipitated opioid withdrawals and/or treatment disengagement. The third was rapid transitioning to an injectable long-acting buprenorphine depot which reduced unnecessary face to face patient contact and treatment non-adherence. This case study of five patients highlights the valuable role that buprenorphine microdosing can play in making induction to long-acting buprenorphine depot feasible to a broader range of patients, including those on a high dose methadone treatment regime.

## Introduction

Scotland has the highest per capita Drug Related Death (DRD) rate in Europe, approaching that of the United States, with opioids implicated in 86% of cases (Christie, 2019). The Drug Deaths Task Force (DDTF) (Scottish Government, 2019) was created by the Scottish Government to stem this rising trend. A key DDTF priority has been to support service innovations which improve access and availability of Medication Assisted Treatment (MAT) for People Who Use Drugs (PWUD). Innovative, flexible and responsive MAT has become even more important during the COVID-19 pandemic, as people who use opioids and other drugs have heightened health and social risks increasing their vulnerability to poor outcomes (EMCDDA, 2020).

## Background

### PWUD Health and Social Issues

Scotland has an ageing cohort of older drug users (over 40 years old), who, through long drug use careers, experience accelerated metabolic ageing and an earlier onset of cardiovascular and respiratory disease (EMCDDA, 2010; Bachi et al., 2017; Matheson, 2017). In particular, high nicotine and cannabis smoking rates, and the use of inhaled heroin and crack cocaine make PWUD particularly vulnerable to the respiratory and cardiovascular complications of COVID-19 (EMCDDA, 2020; Volkow, 2020; Yang and Jin, 2020). Many PWUD therefore come into the category of people needing to shield for a prolonged period of time during pandemic peaks (Clark et al., 2020) which has implications also for their ongoing mental health (Mental Health Foundation, 2020). Opioids contributed to 86% of DRD in Scotland in 2018–nearly always alongside other drugs and/or alcohol (Scottish Government, 2019).

There is a higher incidence of Hepatitis C Virus (HCV) and Human Immunodeficiency Virus (HIV) among people who inject drugs (PWID) (Larney et al., 2017) and an increased likelihood of this rising during the pandemic (EMCDDA, 2020). This is due to a combination of riskier drug use as usual supplies dry up and reduced access to injecting equipment, blood borne virus testing and MAT as already beleaguered addiction services (Larney et al., 2017) face additional challenges from the pandemic such as maintaining continuity of care while protecting frontline workers (Farhoudian et al., 2020; Radfar et al., 2020).

Finally, PWUD often experience greater exclusion and isolation, family separation, unstable housing or homelessness and imprisonment. These factors alongside an increased risk of withdrawals in the absence of access to MAT mean that PWUD would struggle to practice social distancing, self-isolation or shielding advice (EMCDDA, 2020), with significant implications for both their own and public health (Farhoudian et al., 2020).

### MAT Service Innovations During the Pandemic

#### Low Threshold and Assertive Outreach MAT Service

In March of 2020, in response to COVID-19 and the first United Kingdom wide lockdown, we initiated an assertive outreach and low threshold drop-in program to enable people with opioid dependence to access evidence based treatment such as buprenorphine and methadone rapidly (Gibson, 2020). In keeping with the literature around low threshold MAT, the service provided same-day treatment entry and prescribing where appropriate, a harm reduction approach, flexibility in terms of appointments, dispensing and re-initiation if a visit was missed (Jakubowski and Fox, 2020). Many of the patients captured by this clinical intervention were older (over 45 years old) with Chronic Obstructive Airway disease (COPD) and other co-morbidities such as HIV or HCV. Many also had unstable housing or were street homeless and some had never been in treatment before (Gibson, 2020).

#### Buprenorphine Microdosing to Enable Same Day Induction Onto Oral Buprenorphine

While the United Kingdom has both methadone and buprenorphine medications as first line options for MAT (Independent Expert Working Group, 2017), there may be an advantage to the latter in terms of its safety profile, although this needs to be balanced against the patient’s own preference and consequent concordance (Kimber et al., 2015). Buprenorphine is a partial μ-opioid agonist, with high receptor affinity and a ceiling effect on respiratory depression. This results in an effective, long-acting treatment for opioid dependency which may be safer in those with compromised respiratory function for example, due to COPD and/or poly-substance use. Our older patients already with increased risk of both chronic heart disease as well as COVID-19 may be more vulnerable to the cardiovascular adverse effects associated with high dose methadone such as QTc prolongation (Independent Expert Working Group, 2017).

Due to buprenorphine’s strong binding affinity for the μ receptor which supersedes that of the majority of full μ agonists, introducing it in opioid dependent patients who are not in withdrawal can induce this intensely unpleasant state (Soyka, 2017). To avoid this happening, current guidance requires the patient to be in moderate withdrawal (Clinical Opioid Withdrawal Scale greater than 13 (Independent Expert Working Group, 2017)) before taking their first dose. If the patient has been taking short acting opioids, this often means abstinence for 12–24 h and 48–72 h for long-acting drugs such as methadone (Independent Expert Working Group, 2017). When switching from high dose methadone, the requirement is more stringent, with prior tapering to 30 mg or less daily and a cessation of at least 36 h before induction. This is a simple process to understand, but difficult for the patient to do and it is associated with destabilization due to an often prolonged methadone taper (Rozylo et al., 2020).

Microdosing, also known as the Bernese method, is the practice of administering minute doses of buprenorphine to obtain benefit from its action with minimal side effects. It was first described in a case report in 2016 (Hämmig et al., 2016) and proved the pharmacological hypothesis that administering small amounts of buprenorphine to an opioid dependent person who is comfortable on their drug of choice, does not precipitate opioid withdrawal. Further, due to its relatively long half-life, buprenorphine gradually accumulates at the opioid receptors ultimately replacing the μ-agonist enabling the patient to cease its use (Hämmig et al., 2016). As a result, this method is particularly useful where:

• Patients have failed or refused a conventional induction due to the inability to tolerate moderate withdrawals and/or for whom opioid withdrawals would be harmful for example when presenting with poor physical or mental health or pregnancy (The College of Physicians and Surgeons of Manitoba, 2020)
• Significant social instability making regular pharmacy and/or clinic attendances difficult such as homelessness or poverty (The College of Physicians and Surgeons of Manitoba, 2020)
• Patients are switching from high dose methadone or other long acting opioid and need a more tailored cross tapering with buprenorphine (McLean, 2018)
• Patients are attending unscheduled care settings such as accident and emergency or low threshold services in crisis, where the provision of written microdosing instructions, a limited supply of medication and clear follow up can serve to engage this high-risk population (The College of Physicians and Surgeons of Manitoba, 2020)

The evidence base supporting microdosing is not extensive and based primarily on case reports and clinical experience. There are a limited number of good practice guidelines produced mainly by Canadian healthcare organizations (McLean, 2018; Saskatchewan College of Pharmacy Professionals, 2020; The College of Physicians and Surgeons of Manitoba, 2020) which have used microdosing extensively where conventional induction methods are not possible and/or practical. Several variations in the original Bernese method are available (Hämmig et al., 2016; Lu and Cho, 2018; McLean, 2018; Klaire et al., 2019; Terasaki et al., 2019; St.Vincent’s Department of Addiction Medicine, 2019; Moe et al., 2020; Rozylo et al., 2020; Saskatchewan College of Pharmacy Professionals, 2020; The College of Physicians and Surgeons of Manitoba, 2020; James et al., 2021) depending on prescriber and/or clinical settings, with starting dosages ranging from 0.2 to 0.5 mg daily. Table 1 outlines some of these regimens ranging from a 7–11-days induction period. In Canada, where most of these regimens originate, it is common practice to use buprenorphine/naloxone combinations which are quartered or halved to make up the smaller initial doses. Some of the twice daily regimens involve the patient having a supervised dose earlier in the day and a takeaway for later in the day.

**TABLE 1:** Examples of various buprenorphine microdosing schedules.

	Day	1	2	3	4	5	6	7	8	9	10	11
1. Bernese method (20)	Dose (mg)	0.2	0.2	0.8 + 2	2 + 2.5	2.5 bd	2.5 + 4	4 bd	4 bd	8 + 4	Titrate PRN	
2. Terasaki et al. (2019) (20)	Dose (mg)	0.5	0.5 bd	1 bd	4 bd	8	8 + 4	12	Titrate PRN			
3. VCH (22)	Dose (mg)	0.25	0.25 bd	0.5 bd	1 bd	2 bd	4 bd	12	Titrate PRN			
4. Lu & Cho (2018) (22)	Dose (mg)	0.5 bd	1 bd	2 bd	3 bd	4 bd	12	16	Titrate PRN			
5. Used in this study	Dose (mg)	0.4	0.4	0.8	1.2	1.6	1.6	2	4	6	8–12	16

VCH, Vancouver Coastal Health bd twice a day. PRN as required.

In microdosing method 1 and 5, the patient tapers down on their full agonist on day 7 to a full stop by day 11. In methods 2,3 & 4, cessation of the full agonist should happen on day 7.

**TABLE 2:** Proposed dosing schedule based on feedback from patients.

Day	1	2	3	4	5	6	7	8	9	10	11	12	13	14
Dose (mg)	0.4	0.4	0.8	1.2	1.6	1.6	2	4	6	6	8	8	16	24

Taper of full agonist occurs on day 7, complete by day 14. The total daily dose of buprenorphine may be taken in two divided doses where the individual is experiencing the onset of withdrawals or is particularly anxious about the possibility.

#### Transitioning to an Injectable Long-Acting Buprenorphine Depot

In August 2019, a depot buprenorphine formulation allowing weekly or monthly subcutaneous injections was approved for use by the Scottish Medicines Consortium for the management of OUD (Scottish Medicines Consortium, 2019). Early on in the onset of the pandemic, the Scottish Government and the Victorian Government in Australia were at the forefront in identifying the potential benefits of making depot buprenorphine more readily available for high risk groups (Scottish Government, 2020; Straub et al., 2020).

The projected benefits of depot buprenorphine included increased protection of frontline workers and patients seeking MAT from droplet spread of COVID-19 by reducing daily or frequent attendances in pharmacies, enabling people who have been asked by the government to self-isolate or shield to be able to do so, reducing the impact upon patients of pharmacies being closed due to illness or quarantine (Chappuy et al., 2020; Straub et al., 2020). Further, depot buprenorphine also negates the risks such as diversion or overdose implicit in allowing larger amounts of takeaway controlled drugs to minimize unnecessary travel, and the efficacy of treatment will no longer be dependent on the patients adherence to daily dosing, resulting in less risk of overdose and withdrawals (Vorspan et al., 2019; Chappuy et al., 2020).

In order to benefit from the buprenorphine depot however, patients need to go through a similar induction processes as for the oral formulation. For example, a public hospital in Victoria, Australia, has launched the first rapid access clinic for depot buprenorphine and suggests that people in need of a transfer from methadone to buprenorphine-based treatment may require admission to a residential withdrawal unit (Straub et al., 2020). Certainly, in our setting, places in such units are hard to come by, costly, and have been suspended since the pandemic started. The Scottish Government produced a document recommending the use of depot buprenorphine in prisons to provide effective protection against withdrawals while also protecting staff and patients from exposure to COVID-19 (Scottish Government, 2020). However, significant numbers of opioid dependent patients in prison are on methadone, and broad acceptance of depot buprenorphine may be limited by the expectation that they should cease their full agonist in order to go into moderate withdrawals before being given their first injection (Scottish Government, 2020).

Similarly, we encountered patients in our clinic who were keen to have depot buprenorphine but would not have tolerated conventional induction. We therefore present a case study of five patients who were identified through our low threshold intervention who were inducted unto a long-acting buprenorphine depot through a microdosing regimen.

### Case Definition

This case study consists of consecutive patients attending an assertive outreach service between March and October 2020, with a confirmed history of opioid dependence who wished to commence depot buprenorphine for whom conventional induction precautions were not or unlikely to be tolerated. Further, all the included individuals went through a tailored microdosing bridging schedule onto an adequate sublingual buprenorphine dose up to the day before the depot formulation was administered. Excluded were individuals who transitioned onto depot buprenorphine via conventional means as described in the manufacturers product information (The electronic medicines compendium, 2018), or individuals who completed a microdosing schedule in order to remain on sublingual buprenorphine, even if they opted for the depot later on in their treatment.

### Cases

Table 3 provides an overview of the five patients seen for microdosing and induction of Buvidal.

**TABLE 3:** Summary of patient characteristics and case histories.

Case Number	Gender	Age (years)	Primary opioid/s daily use	Reason for patient selection	Microdosing regime start to first depot (Days)	Depot buprenorphine dose and frequency
1	M	36	Methadone 75 mg Prescribed	Treatment failure with sublingual buprenorphine, methadone and naltrexone implant	14	96 mg monthly
2	M	45	Heroin 0.5–1 g inhaled	Treatment failure on both sublingual buprenorphine and methadone. Required to shield due to severe COPD.	19	96 mg monthly
3	F	51	Heroin 0.5–1 g snorted	Treatment failure on both sublingual buprenorphine and methadone. Frequent disengagement from services due to employment. Required to shield due to severe COPD.	8	96 mg monthly
4	M	42	Heroin IV 1 g and methadone 80 mg prescribed	Treatment failure with methadone and with residential rehabilitation. Multiple deliberate and accidental overdoses. Due to polysubstance use and pandemic restrictions, struggled with regular pharmacy attendance. HIV positive with ongoing IVDU.	13	128 mg monthly
5	M	46	Heroin IV 1 g, and methadone 40 mg prescribed	Treatment failure with methadone. Frequent episodes of acute renal colic resulting in a need for A/E admission and analgesia, disrupting dose collection at pharmacy. Difficulty in ceasing IVDU. HIV positive	13	128 mg monthly

M- Male F- Female. COPD- Chronic Obstructive Pulmonary disease. HIV- Human Immunodeficiency Virus. mg-miligram. g-grams. IVDU- intravenous drug use.

#### Case 1 Referred to Here as John (Male, 36 Years Old) had a Long History of IVDU, From the Age of 14 years

He is HIV positive and struggled at different times with alcohol dependence, crack cocaine, heroin and illicit benzodiazepine use. Through the years, John had been on methadone and buprenorphine and managed to stabilize for periods of time, but inevitably struggled with attendances at the pharmacy. He also became criminally involved when intoxicated. He self-funded a naltrexone implant, a treatment modality not offered in Scotland which helped him stay of opioids for around 1 month. He seemed to feel an effect from heroin use which made him wonder if the implant had been inauthentic.

John was on methadone 75 mg daily and was keen to convert to depot buprenorphine so as to cease regular pharmacy pick-ups. We started John on the 14-days regimen with at home microdosing with regular telephone support. John was provided with 15 × 0.4 mg, 9 × 2 mg and 4 × 8 mg sublingual buprenorphine tablets and clearly color-coded instructions. We agreed that John would reduce his methadone to 70 mg at the outset and would then taper down further on his methadone doses once he was on 4 mg of buprenorphine. On his eighth day, we began to taper down by 10 mg daily and he ceased all methadone when he got to 16 mg buprenorphine. John managed the regimen with no issues, and on the 14th day we administered the buprenorphine depot as a 96 mg monthly dose.

#### Case 2 & 3 Referred to Here as Derek (Male, 45 Years Old) and Eleanor (Female, 51 Years Old)

Derek and Eleanor are a married couple. Eleanor often disengaged from OST when pharmacy attendances interfered with her employment. Derek was entrenched within the local drug-using scene and had been criminally involved. When the pandemic started, the couple was required to shield for three months due to underlying COPD. They were both finding that their substance use was having a significant impact on their respiratory function and wanted to stop. Eleanor opted for depot buprenorphine first while Derek was more dubious. Both were concerned about the risk of precipitated withdrawals, something they had experienced in the past.

Eleanor struggled somewhat to understand the microdosing instructions, especially differences in the tablet strengths. We dispensed the 0.4, 2 and 8 mg tablets to her only when they were due to be initiated. With appropriate pandemic related precautions, we administered a 96 mg s/c monthly depot buprenorphine injection at her home once she settled on a 16 mg s/L dose. Eleanor was pleased with the outcome of her treatment and her experience encouraged Derek to undergo the same process. We started his microdosing regime and scheduled Derek’s first injection of the same dose on the day of Eleanor’s second injection. Follow up reviews of the couple have been positive.

#### Case 4 & 5 Referred to Here as George (Male, 42 Years Old) and Harry (Male, 46 Years Old)

George and Harry have been a couple for over 3 years. Both are HIV positive on anti-retroviral medications. George had a much longer history of IV heroin use, and a long and varied treatment experience which included periods on prescribed methadone, buprenorphine and also two periods in an abstinence-based recovery program. George also had a 20-years history of benzodiazepine use, initially through a prescription, but latterly from the illicit market. Harry had a much shorter history of IV heroin use and has always needed George to inject him. Harry had never been on any form of MAT. Both attended for treatment at the same time when George was discharged from his rehabilitation program due to the pandemic. They opted to be seen together and requested methadone. Unfortunately, after two non-fatal overdoses, it was clear that methadone was not reducing their risk.

We discussed buprenorphine and both were concerned that they would struggle with concordance. Also, as George was on 80 mg of methadone, he was concerned that he would not manage the associated withdrawals of conventional induction. We needed to specifically counsel Harry around the reduced efficacy of opiate analgesics should he need to attend the accident and emergency (A&E) department with renal colic which he sometimes suffered. He was reassured however by our standard practice of placing a medical alert in shared records about patients being on depot buprenorphine. In his case, should he attend A&E in acute pain, hospital care staff will recognize that he will need larger doses of opioid analgesics or alternate analgesics altogether.

Both patients stabilized on 24 mg of s/l buprenorphine which translated into 128 mg of monthly the depot which was administered in the clinic. We were particularly concerned about George and Harry’s illicit benzodiazepine use. It was unrealistic to expect them not to take some benzodiazepines, especially for George who had a twenty-year history of dependence on these. There is a known risk of benzodiazepines reducing the ceiling effect of buprenorphine, such that combining these with other drugs may result in an overdose. Once this happens, higher naloxone doses would be required due to the high receptor affinity that buprenorphine has. We agreed on a maintenance dose of 20 mg a day of diazepam on an interval dispensing regime to support them in trying to avoid the far more potent illicit benzodiazepines known to be circulating in the local market. Also, they were provided with further naloxone kits and ongoing support. On the latest review, both have fully ceased IV drug use and have managed to avoid illicit benzodiazepines.

## Discussion

As a confluence of three service delivery innovations, this case study is an example of a nimble response to unprecedented challenges to addiction services. Also, to our knowledge, this is the first case study describing the use of a microdosing regimen to induct patients with opioid use disorder unto a long-acting buprenorphine depot. This study is limited by its retrospective case study design, the absence of a comparison group, short duration of follow up, and a lack of objective outcome measures, such as systematic urine results. Furthermore, assessment of withdrawal was by clinician impression and patient self-report as opposed to a formal Clinical Opiate Withdrawal Score (COWS) (Avery and Taylor, 2019). This is partly as, in a less than ideal setting of a time limited outreach visit, formally completing a COWS can be challenging. Nevertheless, objective measures such as these would have made cross-comparisons across different clinical settings more feasible.

Furthermore, each of the three innovations came with its challenges. For example, while a low threshold assertive outreach model improves access to marginalized groups (Bond and Drake, 2015; Hurley and O’Reilly, 2017), robust clinical governance must be in place to ensure the patient’s medical and medications history is known before a prescription is initiated and to avoid duplicate prescribing of controlled drugs. Inevitably, there will be times that a systems failure results in delays in MAT initiation and consequently, the possibility of patient disengagement.

While buprenorphine microdosing has clear advantages in over-coming potential delays inherent in traditional induction on the day or patient presentation, it is important to note that it cannot be recommended as an equivalent alternative to current standard practice due to the lack of high level evidence such as randomized controlled trials. There have however been case reports and substantial practical experience with this method in Canada, Germany and locally in the South East and the West of Scotland (Cassells et al., 2020). The result is a broad range of regimens with no consensus on optimum dosing.

Conventional induction and stabilization unto buprenorphine is attainable within 2–3 days provided the patient is able to tolerate withdrawals. Microdosing can take 7–14 days with the patient continuing to use illicit opiates as required. Microdosing therefore increases immediate accessibility to buprenorphine but prolongs the patient’s risk exposure to illicit drug use by several days. Finally, in North America particularly, the use of buprenorphine/naloxone combinations are favored over buprenorphine on its own This is primarily to avoid situations of diversion or misuse of buprenorphine for example through snorting or injecting it. The consequence of this is that the smaller doses within a microdosing regime (less than 2 mg) usually consists of portions of buprenorphine/naloxone tablets. These tablets are used sublingually and so are friable, meaning that the dosing accuracy is likely to be variable (Rozylo et al., 2020). Our strategy has been to use buprenorphine sublingual tablets available in 200 and 400 mcg doses. This has allowed us to provide more accurate dosing and simpler patient instructions.

While the pandemic highlighted the advantages of depot treatment in terms of reducing the risks of exposure to COVID-19, there are definite limitations that need to be considered. These include the need for nursing or medical staff to administer the injection (Scottish Medicines Consortium, 2019), the significantly higher costs (20% higher than oral formulations, 16 times more expensive than methadone solution) (Scottish Public Health Observatory, 2019), the lack of generic products to compensate for potential supply chain interruptions and limited clinician and patient experiences with its use. Further, the consumption of large amounts of potent street benzodiazepines, a particularly worrying issue among PWUD in Scotland, reduces the threshold of the ceiling effect of buprenorphine, removing the protection patients normally have against respiratory depression (Independent Expert Working Group, 2017). Once administered, the depot injection dose cannot be removed and the long duration of action of buprenorphine magnified by its prolonged release formulation will limit the effectiveness of naloxone (Chappuy et al., 2020). Unfortunately, the extent to which this scenario is likely to increase patient risk is as yet poorly understood.

Buprenorphine makes up 19% of MAT for opioid use disorder in Scotland, with the remainder being primarily methadone (Scottish Public Health Observatory, 2019). Clinical experience in Scotland is that patients tend to opt for methadone, possibly as this is what they are more familiar with. During the pandemic, and within the context of our outreach model of care, it was often clinically safer to encourage the use of buprenorphine. It may be that with the introduction of the depot, more flexibility in induction through microdosing and increased patient education as to its favorable safety profile, buprenorphine may become increasingly more common.

What we have been able to demonstrate is a range of clinical scenarios where microdosing has been effective in inducting patients onto depot buprenorphine enabling them to gain from the benefits of this treatment at a crucial time. We have also been able to administer depot buprenorphine injections to patients in their homes, supporting them to adhere to government advice on shielding. Notably, some of our patients sometimes found the microdosing regimen confusing and starter packs or pre-prepared dosette boxes of buprenorphine tablets used in some settings (Terasaki et al., 2019) could be a helpful addition to our practice.

Issues which need to be better understood include a cost benefit and sustainability analysis based on a larger number of cases. Specifically, will investment in the more expensive depot buprenorphine injection reduce the available resources to provide care for the growing number of people who use drugs needing treatment? Further, what are the implications for the patient when their treatment is distilled into a monthly visit for an injection? Indeed, with an eye on the adaptations we undertook to provide ongoing care for patients during the pandemic, it is also important to evaluate what the essential components of safe and high-quality MAT actually are. In other words, what aspects of our practice in initiating buprenorphine and methadone must be continued for the safety of our patients, and what aspects simply represent organizational dogmatism?

This last point relates to the need to develop the quality of the evidence base around microdosing. At what point do we acknowledge the successful application of clinical expertize over many years in the application of buprenorphine microdosing, almost a naturalistic clinical trial, rather than insisting on interventional randomized controlled trials? Perhaps if it is randomized controlled trials which are required, the possibility of using microdosing as a means to induct unto the relatively newly developed range of long-acting buprenorphine depot injections may provide the necessary impetus.

## Conclusion

The COVID-19 pandemic is challenging health systems throughout the world and forcing addictions services to be agile and innovative to meet the needs of PWUD while also protecting them and frontline workers from viral transmission. This study demonstrates the utility of using microdosing to facilitate the induction of patients onto depot buprenorphine in situations where conventional methods are impractical or not tolerated. Certainly, microdosing may be a more affordable and acceptable alternative to an inpatient detoxification or subjecting patients on high dose methadone to unpleasant withdrawals as is currently practiced in some settings. The lack of published literature on buprenorphine microdosing undertaken in a range of different settings is a barrier to its more widespread adoption. We propose an international collaboration to collate clinical experience and case report data and produce definitive best practice guidelines in the mainstream use of buprenorphine microdosing.

### Permission to Reuse and Copyright

Figures, tables, and images will be published under a Creative Commons CC-BY license and permission must be obtained for use of copyrighted material from other sources (including re-published/adapted/modified/partial figures and images from the internet). It is the responsibility of the authors to acquire the licenses, to follow any citation instructions requested by third-party rights holders, and cover any supplementary charges.

## Data Availability

The original contributions presented in the study are included in the article/Supplementary Material, further inquiries can be directed to the corresponding author.
